# Computational Discovery of Transcriptional Regulatory Modules in Fungal Ribosome Biogenesis Genes Reveals Novel Sequence and Function Patterns

**DOI:** 10.1371/journal.pone.0059851

**Published:** 2013-03-29

**Authors:** Viktor Martyanov, Robert H. Gross

**Affiliations:** 1 Department of Genetics, Geisel School of Medicine at Dartmouth, Hanover, New Hampshire, United States of America; 2 Department of Biological Sciences, Dartmouth College, Hanover, New Hampshire, United States of America; University of California Riverside, United States of America

## Abstract

Genes involved in ribosome biogenesis and assembly (RBA) are responsible for ribosome formation. In *Saccharomyces cerevisiae*, their transcription is regulated by two dissimilar DNA motifs. We were interested in analyzing conservation and divergence of RBA transcription regulation machinery throughout fungal evolution. We have identified orthologs of *S. cerevisiae* RBA genes in 39 species across fungal phylogeny and searched upstream regions of these gene sets for DNA sequences significantly similar to *S. cerevisiae* RBA regulatory motifs. In addition to confirming known motif arrangements comprising two different motifs in a set of *S. cerevisiae* close relatives or two instances of the same motif (that we refer to as modules), we have also discovered novel modules in a group of fungi closely related to *Neurospora crassa*. Despite a single nucleotide difference between consensus sequences of RBA motifs, modules associated with *S, cerevisiae* group and *N. crassa* group displayed consistently different characteristics with respect to preferred module organization and several other module properties. For a given species, we have found a correlation between the configuration of the RBA module and significant enrichment in a set of specific Gene Ontology biological processes. We have identified several likely new candidates for a role in ribosome biogenesis in *S. cerevisiae* based on the combined evidence of RBA module presence in the upstream regions, functional annotation information and microarray expression profiles. We believe that this approach will be useful in terms of generating hypotheses about functional roles of genes for which only fragmentary data from a single source are available.

## Introduction

According to the Gene Ontology (GO) [1], the biological process category of ribosome biogenesis and assembly (RBA) includes genes involved in the biosynthesis, assembly and arrangement of ribosome components as well as in the transport to protein biosynthesis sites.

The question of the transcriptional regulation of fungal genes involved in RBA has received a great deal of attention given the paramount importance of this functional category. It has been shown that RBA genes are regulated differently from the actual ribosomal proteins that are the physical constituents of ribosomes [2], [3]. In *Saccharomyces cerevisiae*, RBA genes were found to have multiple instances of two characteristic upstream DNA regulatory motifs [4]: the A/T-rich ribosomal RNA processing element (RRPE) [5] and the polymerase A and C (PAC) box with a specific GATGAG consensus [6]. Binding factors have been identified for these DNA sequences: Stb3 for RRPE [7] and Dot6/Tod6 for PAC [8]–[10].

Multiple authors have observed the interesting characteristics of the RRPE and PAC arrangement in *S. cerevisiae*, including significant biases in the spacing between the two motifs, distance to the translation start site, and order and orientation of the individual motifs [11]–[16]. It has also been shown that these motifs are conserved across other fungi closely related to budding yeast [17], [18] and that this regulatory pattern is unique to fungi, in comparison to other eukaryotes [19].

Here we present some novel aspects of the RBA regulatory signals. While previous papers primarily dealt with the close relatives of *S. cerevisiae*, we considerably expand the species selection by adding dozens of other fungi distributed across fungal phylogeny. We also design and apply a comprehensive computational approach aimed at identifying the highest-scoring combinations of putative regulatory DNA motifs in a set of genes. Using this approach, we verify and refine previously reported patterns and discover novel motif arrangements (called modules), which allow us to hypothesize about the evolution and conservation of RBA regulation across all major fungal phyla. We also discover a possible relationship between the organization of a module and the enrichment in particular GO biological processes for RBA gene set in a given species and demonstrate that module information can be used to discover new genes putatively involved in RBA.

Starting with the list of *S. cerevisiae* RBA genes, we have identified orthologs in 38 other fungal species. We then analyzed upstream regions of the RBA genes in each species by using SCOPE [20], an ensemble motif finder. From each SCOPE run, we identified the best matches to the biologically verified RRPE and PAC sequences and analyzed the positional distribution of these motifs in the upstream regions. Further computational analysis of the SCOPE data led to the identification of high scoring pairs of motifs (modules). Using this approach, we showed the existence of several distinct and well defined modules across the fungal kingdom involving RRPE and/or PAC, which substantially complements and expands previous knowledge in the field. The module patterns showed a distinct behavior for two different groups of closely related fungal species. For several species, we found a relationship between motif order within a module and biological functions of the corresponding genes. We were able to use module information to find new candidate genes that are likely to be involved in the RBA biological process.

## Materials and Methods

### *S. Cerevisiae* Gene Set Retrieval and Fungal Ortholog Generation

We retrieved the starting RBA gene set from the Saccharomyces Genome Database (SGD) [21]. For other fungi, we used fungal phylogeny based on 153 universal orthologs in 42 fungal species with completely sequenced genomes [22]. Thirty-eight fungal species (in addition to *S. cerevisiae*) were analyzed in our study.

Fungal orthologs for *S. cerevisiae* targets of RRPE and PAC were identified by mining PhylomeDB [23], a public repository of complete collections of gene phylogenies. When the internal PhylomeDB identifiers were not readily convertible to standard gene names, we used the RSAT suite [24] to find reciprocal corresponding best matching orthologs.

### Motif Comparison

We first used STAMP [25] to calculate the similarity between *S. cerevisiae* computationally predicted motifs (from SCOPE) and RRPE and PAC sequences as reported in [17]. We then calculated similarities between these best-matching SCOPE motifs for *S. cerevisiae* and high-scoring motifs from each SCOPE run of corresponding orthologs in other fungal species. The *S. cerevisiae* motif served as an input motif and was compared to a user-defined dataset of SCOPE motifs from each fungal ortholog run. This way, we compared motifs that were generated via the same approach (i.e. by doing SCOPE runs). For each comparison of the *S. cerevisiae* motif to another fungal species motif, a p-value was calculated reflecting a degree of sequence similarity between two motifs. STAMP analysis was run with default alignment parameters: Pearson Correlation Coefficient for column comparison metric, ungapped Smith-Waterman for alignment method, iterative refinement for multiple alignment strategy and UPGMA for tree-building algorithm.

### Motif Pattern Analysis

In addition to identifying individual upstream sequences, we were also interested in analyzing patterns of motifs (or modules). Our current working definition of a module is the arrangement of two motif instances separated by a conserved distance and having a conserved organization.

In order to distinguish between different modules, we have developed a module score that reflects the overall quality of a module. The module score is comprised of five different metrics:

1. Sig value, a measure of the statistical significance of each component motif as calculated by SCOPE. It is a measure of the overrepresentation and position distribution of a motif in a given gene set compared to the rest of the genome;
2. ABC score (Area Between Curves), a measure of the non-randomness of the distribution of inter-motif distances for a module. It is calculated by comparing the inter-motif distance distribution for the actual modules to the inter-motif distance distribution for modules that are generated by shuffling the same number of instances of component motifs. ABC score ranges from −1 to +1 and its negative value indicates that the motifs in actual modules are further apart than expected by chance alone whereas positive ABC scores indicate that the motifs in actual modules are closer than expected by chance alone;
3. Module position, a measure of the non-randomness of the distribution of the upstream position of a module for the entire set of module occurrences;
4. Coverage, the fraction of genes in the starting gene set that contain the module of interest;
5. Motif orientation within modules, a measure of the preferred orientation of motifs within a module with respect to DNA strand (both motifs in the same orientation vs. motifs in different orientation).

These statistics are combined into a module score using weights that can be adjusted by the user. Module score is normalized to the range of 0 to 1 with higher module scores meaning that a given motif pattern is more likely to be important for transcriptional regulation.

Module analysis utilizes newly developed algorithms and statistical analyses that generate a module score. It is conducted with a new program we developed called Module Locator (manuscript submitted). Module Locator takes SCOPE-generated XML output files and examines and evaluates all combinations of motifs that might be of interest. Once the paper is accepted for publication, it will be freely available for both Macintosh and Windows platforms. It contains extensive help section and a sample XML file for module analyses.

### Comparison of Module Attributes across Fungal Species

In order to analyze the behavior of module characteristics across fungal groups, we have created a series of box plots. For a given group of species and a given statistic, the box plot allows easy visualization of the range of data values within the group.

Since our implementation of this method does not deal with negative values, we adjusted the input for ABC score by adding 1 to all data values to make its range from 0 to 2 (instead of −1 to +1). For median upstream position, we used the absolute values indicating how far upstream a module is.

### Functional Enrichment Analysis

The functional annotation tool FunCat [26] was used to calculate functional enrichment in RBA gene sets and their orthologs. It was used for *S. cerevisiae*, *Neurospora crassa* and *Fusarium graminearum*.

### Prediction of New Candidate Genes Involved in RBA

SCOPE has a feature that allows finding additional genes in the genome that have a specified motif and improve its Sig value (the measure of statistical significance of a motif), when added to the original gene set. This sequence information (identification of genes with relevant motifs and modules) represents a first component of our new gene categorization approach. The other two components utilize functional annotation and gene expression data. For *S. cerevisiae*, functional annotation is retrieved from SGD [21] and gene expression information is retrieved from SPELL [27], a search engine that, given the input gene set, finds the most relevant microarray experiments and within them the genes most similar to the input set in terms of the expression profiles.

## Results

### SCOPE Analyses of Fungal Orthologs of *S. Cerevisiae* Genes Involved in RBA

**Figure 1:**
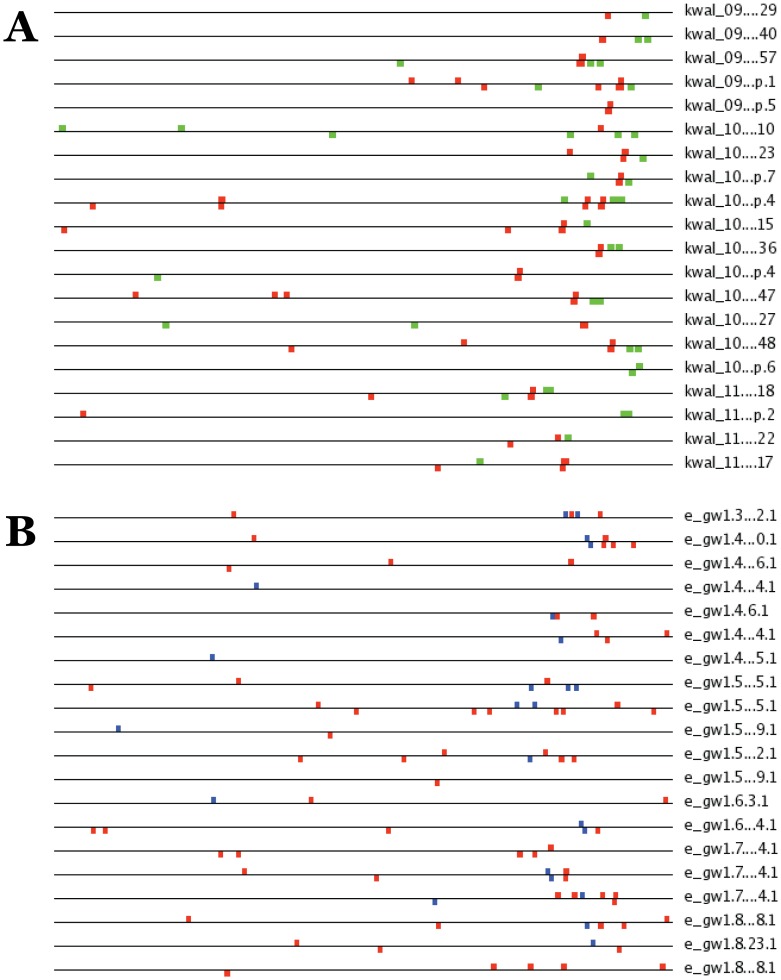
Sample SCOPE outputs of fungal RBA gene runs. (A) SCOPE results for *Kluyveromyces waltii* RBA genes. RRPE – red, PAC – green. (B) SCOPE results of *Trichoderma reesii* RBA genes. PAC2– blue, RRPE – red.

SCOPE runs showed two major kinds of motif combinations: RRPE-PAC and PAC2-RRPE (Figure 1). In each case, the motif order corresponds to the preferred module orientation with respect to the transcription start site, 5′-3′. The RRPE-PAC pattern has been reported previously. PAC2 is a novel observation and a variation of the canonical PAC sequence (GATGAG) that is different in a single nucleotide (GAT**A**AG).

In terms of STAMP comparisons to known RRPE and PAC sequences, we found high-scoring matches to either (or both) motifs in all fungal species in the study (Table S1). The median statistics for all the motifs were a SCOPE Sig value of >240 (which roughly corresponds to a p-value of 1/2^240^ ∼ 5.7×10^−73^), coverage above 70% and a STAMP alignment p-value of 6.5×10^−9^, reflecting a high similarity between motifs identified by SCOPE and canonical RRPE and PAC sequences. SCOPE identified PAC matches in numerous species in which they had not been reported previously.

### Analysis of Positional Distributions of Putative RRPE and PAC Motifs

**Figure 2:**
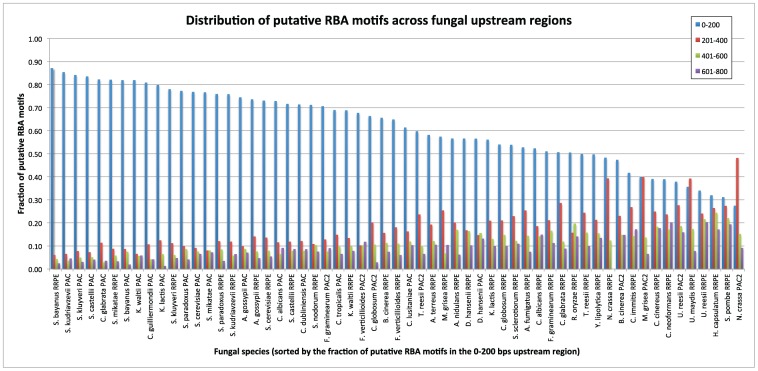
Position distribution of putative RRPE and PAC/PAC2 motifs in fungal upstream regions. X-axis shows RRPE and/or PAC/PAC2 motifs in each fungal species and y-axis shows fraction of occurrences in four different upstream quartiles for each motif.

We partitioned each fungal upstream region analyzed by SCOPE (default length 800 bps) into four quartiles and calculated the fraction of RRPE and/or PAC motifs in each quartile. According to mean statistics, 62% of occurrences of RRPE, PAC or PAC2 were in the 0–200 bps quartile (Table 1). PAC was different from both RRPE and PAC2 as 76% of PAC motifs were in the first quartile, compared to 52% for PAC2 and 57% for RRPE. Out of 59 matches to RBA motifs across all species, 56 (95%) had most instances in the 0–200 bps quartile (Figure 2).

**Table 1:** Distribution of occurrences of RRPE and PAC motifs across four upstream quartiles of orthologs of *S. cerevisiae* RBA genes.

Motif quartiles	Mean for all RRPE and PAC	Mean for RRPE	Mean for PAC	Mean for PAC2
0–200	0.62	0.57	0.76	0.52
201–400	0.18	0.20	0.11	0.26
401–600	0.11	0.13	0.07	0.12
601–800	0.09	0.10	0.06	0.10

Mean value represents average fraction of occurrences across all fungal species for a given quartile region.

### RRPE and PAC Motif Sequence Conservation across Fungal Phylogeny

**Figure 3:**
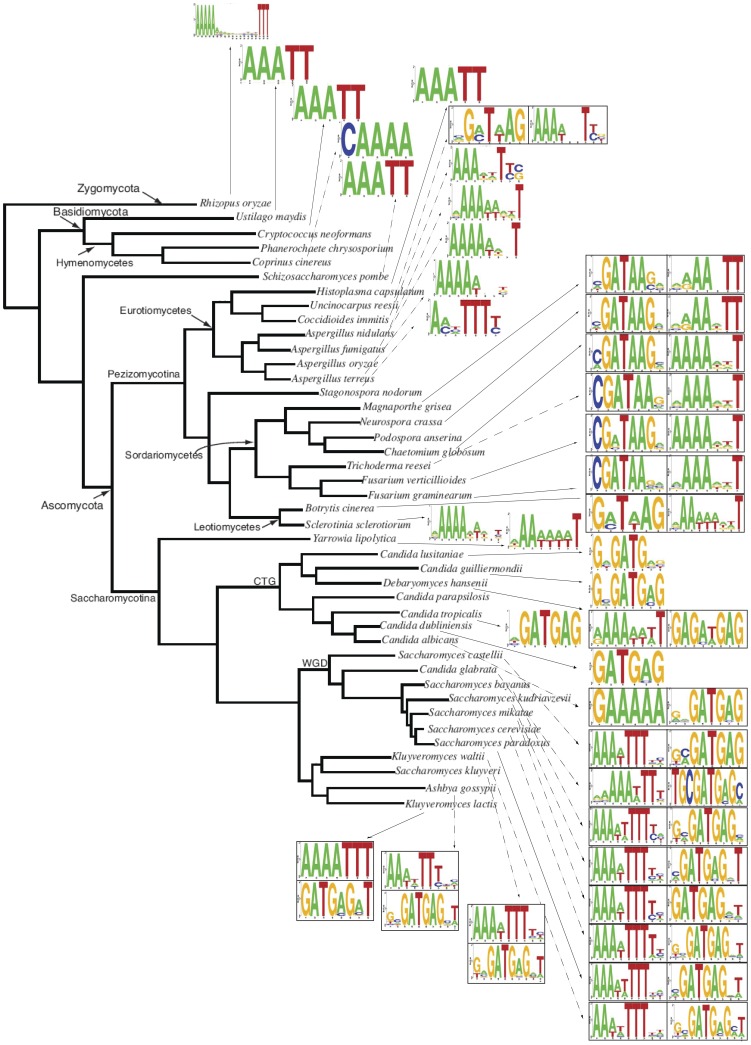
Distribution of the best RRPE and PAC/PAC2 matches across fungal phylogeny. Sequence logos represent the best SCOPE matches to RRPE and PAC/PAC2 sequence for each species.

Figure 3 shows the distribution of RRPE and PAC motif patterns across fungal evolution. RRPE appears primarily in Zygomycota and Basidiomycota and in some Ascomycota fungi. We observe the PAC motif in several *Candida* species. The modules formed by two different motifs correspond to two different groups of non-overlapping fungi. The RRPE-PAC is found in the *Saccharomyces* genus and other related fungi. PAC2-RRPE is present in fungi generally similar to *N. crassa*, corresponding to *Sordariomycetes* and *Leotiomycetes* classes. Table 2 summarizes these data.

**Table 2:** Summary of individual motifs and motif patterns in upstream regions of RBA genes.

Fungal groups	Pattern
Zygomycota, Basidiomycota and some Ascomycota (*Eurotiomycetes*)	RRPE only
Some Ascomycota (*Candida*)	PAC only
Some Ascomycota (*Saccharomyces*, *Kluyveromyces*)	RRPE + PAC
Some Ascomycota (*Sordariomycetes*)	PAC2 + RRPE

### Comparison between Two Main Fungal Groups in Terms of Module Attributes

We selected *S. cerevisiae* and *N. crassa* as two representative species from their respective groups. For both species, we then compared different components of the module score for two module orientations: RRPE-PAC vs. PAC-RRPE for *S. cerevisiae* and RRPE-PAC2 vs. PAC2-RRPE for *N. crassa*.

**Figure 4:**
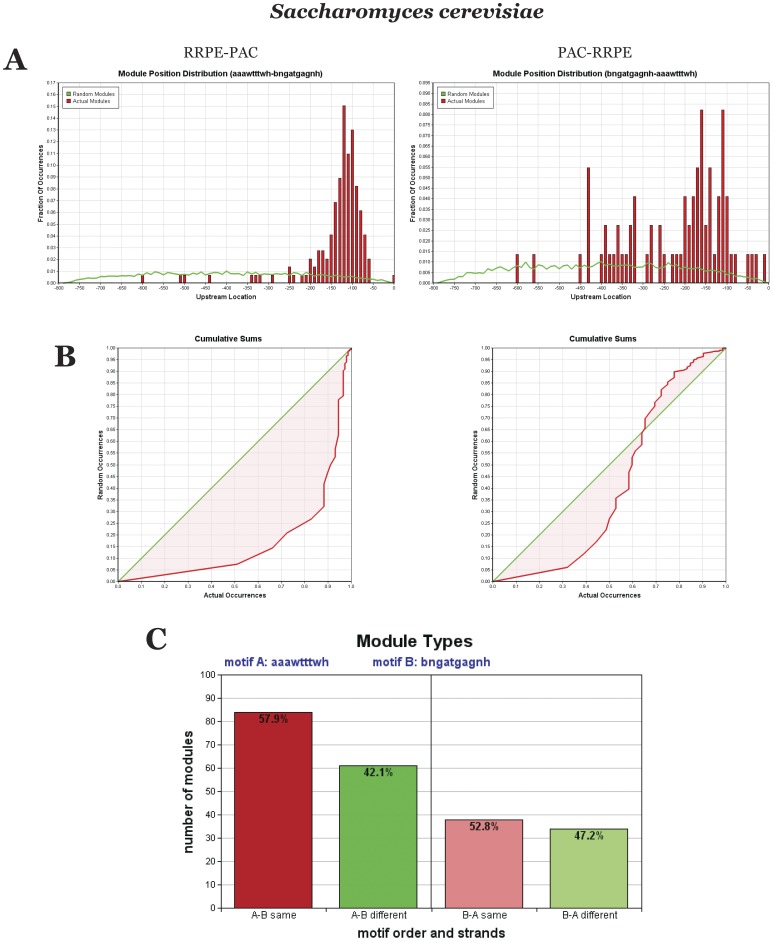
Comparison of two different RBA module organizations in *S. cerevisiae*. (A) Upstream module position distribution. (B) ABC score (inter-motif distance distribution). (C) Number of occurrences and strand preference.

Figure 4 shows the results of Module Locator analyses of *S. cerevisiae* RBA genes. Modules in the RRPE-PAC orientation (the left side of the figure) have a more narrow upstream position distribution (and are located closer to the transcription start site), higher ABC score (reflecting that RRPE and PAC are closer together than by random chance) and higher number of occurrences than modules in PAC-RRPE orientation. Also, in the case of RRPE-PAC, there is a preference for RRPE and PAC to be on the same strand that is not seen for PAC-RRPE.

**Figure 5:**
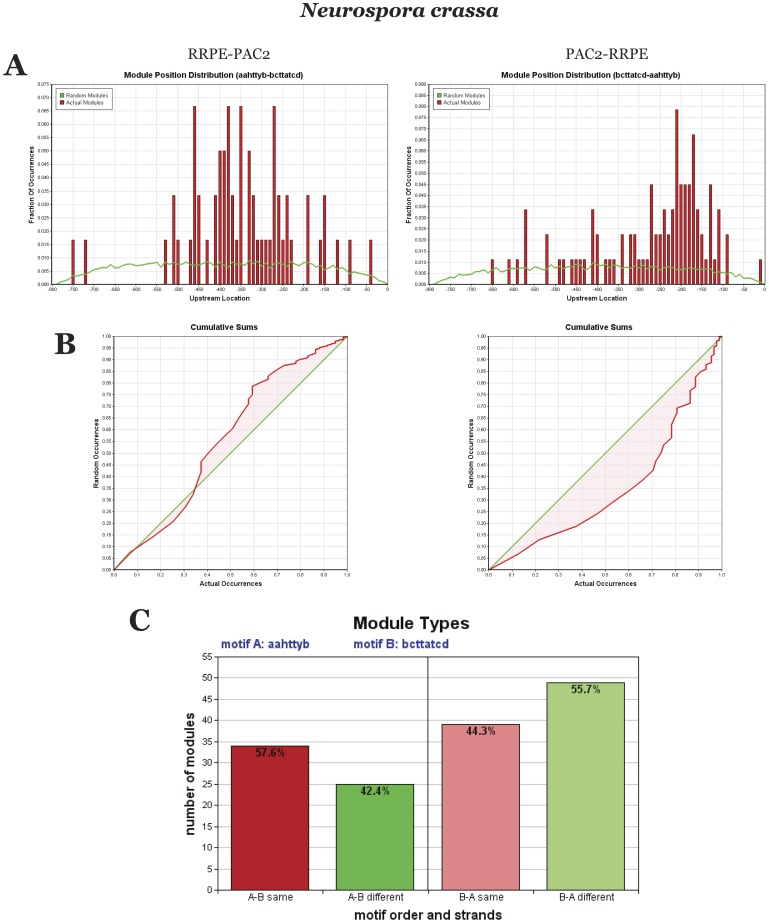
Comparison of two different RBA module organizations in *N. crassa*. (A) Upstream module position distribution. (B) ABC score (inter-motif distance distribution). (C) Number of occurrences and strand preference.

Figure 5 shows corresponding data for *N. crassa* RBA genes. Modules in RRPE-PAC2 tend to have more dispersed upstream position distribution (and are farther away from the transcription start site), lower ABC score (its negative value reflects that RRPE and PAC2 are more distant from each other than by random chance) and lower number of occurrences than modules in PAC2-RRPE order. In terms of the strand preference, modules in RRPE-PAC2 orientation tend to have RRPE and PAC2 on the same strand, whereas modules in PAC2-RRPE order more often have PAC2 and RRPE on different strands.

We have performed these analyses for other fungi in which we observed module patterns. Table S2 summarizes all data for module statistics.

**Figure 6:**
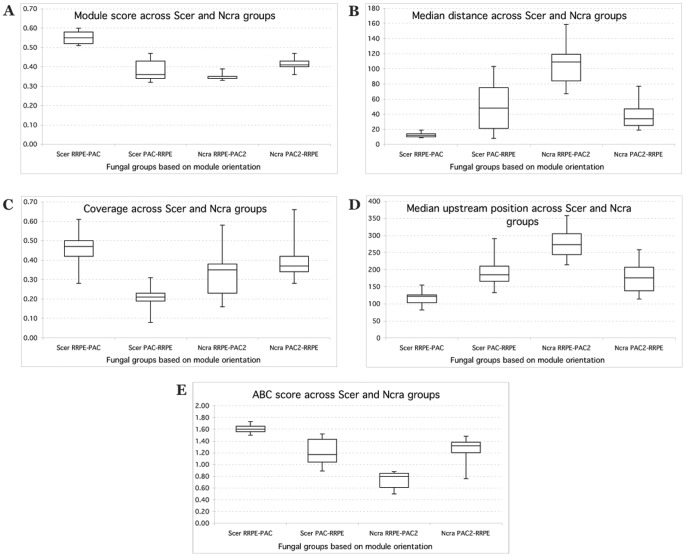
Comparison of RBA module attributes across fungal groups based on module organization. X-axis shows fungal groups (species similar to either *S. cerevisiae* or *N. crassa*) each separated into two subgroups based on two possible organizations of the RBA module. Y-axis shows a specific module attribute [see Materials and Methods for explanation of y-axis values in (C) and (D)]: (A) module score, (B) median inter-motif distance; (C) module coverage; (D) module median upstream position; (E) module ABC score.

In order to visualize the differences between and within two main fungal groups in terms of module characteristics, we constructed several box plots. Figure 6 shows these graphs for several module attributes. RRPE-PAC has a much higher module score than PAC-RRPE whereas RRPE-PAC2 has a lower module score than PAC2-RRPE. RRPE-PAC has a smaller inter-motif distance compared to PAC-RRPE while in the case of RRPE-PAC2 motifs are much farther apart than for PAC2-RRPE. Module coverage for RRPE-PAC is much higher than for PAC-RRPE and it is about the same for both RRPE-PAC2 and PAC2-RRPE. RRPE-PAC modules are closer to the transcription start site than PAC-RRPE modules and the reverse is true for RRPE-PAC2 and PAC2-RRPE modules. Finally, RRPE-PAC has a higher ABC score compared to PAC-RRPE while both orientations have primarily positive ABC scores. In the case of RRPE-PAC2, it has a negative ABC score while PAC2-RRPE has a much higher positive ABC score. Table 3 summarizes the comparisons between fungal groups and shows consistent patterns across closely related species from the same group (either *S. cerevisiae*-like or *N. crassa*-like) as well as consistent differences between two groups in terms of different features of RBA modules.

**Table 3:** Comparison of *N. crassa*-like (Ncra-like) and *S. cerevisiae*-like (Scer-like) fungal groups in terms of RBA module orientations across several module attributes.

Module attribute	Fungal group	Module orientation	Mean value
Module score	Scer-like	RRPE-PAC	0.55
	Scer-like	PAC-RRPE	0.38
	Ncra-like	RRPE-PAC2	0.35
	Ncra-like	PAC2-RRPE	0.42
Median distance	Scer-like	RRPE-PAC	12
	Scer-like	PAC-RRPE	50
	Ncra-like	RRPE-PAC2	106
	Ncra-like	PAC2-RRPE	38
Coverage	Scer-like	RRPE-PAC	0.46
	Scer-like	PAC-RRPE	0.2
	Ncra-like	RRPE-PAC2	0.33
	Ncra-like	PAC2-RRPE	0.4
Upstream position	Scer-like	RRPE-PAC	−118
	Scer-like	PAC-RRPE	−195
	Ncra-like	RRPE-PAC2	−277
	Ncra-like	PAC2-RRPE	−178
ABC score	Scer-like	RRPE-PAC	0.6
	Scer-like	PAC-RRPE	0.2
	Ncra-like	RRPE-PAC2	−0.26
	Ncra-like	PAC2-RRPE	0.26

Mean value corresponds to the average statistic for a given module attribute across a set of closely related fungi.

### Relationship between Motif Order in a Module and Gene Function

In addition to comparisons between motif orders for different module statistics, we also looked at the possible association between motif order and a set of distinct biological functions.

We separated the original *S. cerevisiae* RBA set into two subsets of genes with modules in either RRPE-PAC or PAC-RRPE order only. We compared functional enrichment of these subsets via FunCat [26]. Table 4 lists the statistically significant functional terms (p-value of functional enrichment ≤10^−3^) found for each subset. Genes with RRPE-PAC modules were generally enriched in ‘RNA-level’ processes (e.g., different kinds of RNA metabolism) while genes with PAC-RRPE modules showed enrichment in ‘protein-level’ processes (e.g., ribosome biogenesis and protein synthesis). We repeated these analyses for *N. crassa* and *F. graminearum* and obtained comparable results (Table S3). For *N. crassa*, RRPE-PAC2 modules were found in genes enriched in ‘RNA-level’ processes and PAC2-RRPE modules were present in genes generally enriched in ‘protein-level’ annotations. The reverse was true for *F. graminearum*: RRPE-PAC2 fraction was enriched in ‘protein-level’ processes while PAC2-RRPE subset showed enrichment in ‘RNA-level’ functional terms.

**Table 4:** Relationship between RBA module orientation and gene functional annotation in *S. cerevisiae*.

Orientation	Unique and enriched functional terms
RRPE-PAC	ATP binding
	nucleic acid binding
	nucleotide/nucleoside/nucleobase binding
	RNA binding
	RNA modification
	RNA processing
	rRNA modification
	rRNA processing
	transcription
	tRNA processing
PAC-RRPE	nuclear membrane
	nuclear transport
	nucleus
	protein synthesis
	protein transport
	ribosomal proteins
	ribosome biogenesis
	RNA transport
	structural protein binding

### Prediction of New RBA-associated Genes Based on Combined Evidence

For *S. cerevisiae*, we used the extra gene feature of SCOPE and identified 12 previously unreported genes containing RBA modules (8 with RRPE-PAC and 4 with PAC-RRPE). By utilizing functional annotation and microarray expression data (see Materials and Methods), we were able to find evidence from at least one other source for 8 genes and from two sources for 4 genes. Based on these analyses, we can hypothesize about several new candidates for a role in ribosome biogenesis in *S. cerevisiae*.

The 4 candidate genes that are most likely involved in RBA (supported as such by SCOPE motif and module data, SGD functional description information and SPELL expression data) are as follows. YDR465C is an arginine methyltransferase that methylates ribosomal protein RPL12. YGR272C is an essential protein required for maturation of 18S rRNA. YPR010C is the second largest subunit of RNA polymerase I. YNL061W is a probable RNA methyltransferase essential for large ribosomal subunit biogenesis. Table 5 describes which genes are supported by which analyses and Table S4 contains more information about all 12 predicted genes.

**Table 5:** List of *S. cerevisiae genes* putatively involved in RBA that are predicted using SCOPE, SGD and SPELL data.

Orientation	SCOPE	SGD	SPELL
RRPE-PAC	YDR465C	YDR465C	YDR465C
	YGR272C	YGR272C	YGR272C
	YPR010C	YPR010C	YPR010C
	YBL039C		YBL039C
	YLR074C		YLR074C
	YOR341W	YOR341W	
	YOR205C		
	YDL129W		
PAC-RRPE	YNL061W	YNL061W	YNL061W
	YDL150W	YDL150W	
	YGR129W		
	YLR008C		

Number of times the same gene name is listed means number of different sources of evidence that it may be involved in RBA.

## Discussion

Analysis of top SCOPE motifs across multiple fungal species showed both previously reported and novel DNA sequences. The new variation of PAC (which we refer to as PAC2) is found primarily in a specific subset of fungi (similar to *N. crassa*), reminiscent of PAC being associated exclusively with close relatives of *S. cerevisiae*.

It has been suggested [16], [17] that the PAC motif is younger than the RRPE motif that represents the mode of RBA transcriptional regulation in more evolutionarily ancient fungal species. While our results generally support this idea, they also indicate important novel patterns across fungal phylogeny. We believe that the PAC lineage in the case of PAC2 in *N. crassa* and related fungi originates earlier than previously thought. A clear PAC2-RRPE module is observed as early as in *Uncinocarpus reesii*, a member of *Eurotiomycetes* class different from *N. crassa* lineage. While PAC2 forms a module with RRPE in *Sordariomycetes*, it is not until later in evolution that PAC seems to replace RRPE in *Candida* species that apparently rely just on the PAC motif for their RBA regulation. The branch of *Saccharomycotina* that underwent whole genome duplication and their close relatives (*Saccharomyces* and *Kluyveromyces* species) use the RRPE-PAC module in order to regulate transcription of the RBA genes.

The two fungal groups that have enrichment in both RRPE and PAC (PAC2) motifs in the upstream regions of their RBA genes have a specific preferred order of motifs within modules. For a given group of closely related fungi, this preferred module orientation can be associated with a better module quality (i.e. higher overall module score, smaller distance between motifs comprising modules, higher module coverage and module position closer to transcription start site). For *S. cerevisiae* and related species, RRPE-PAC is the preferred order that always has better quality than its counterpart PAC-RRPE. This rule is exactly reversed for *N. crassa* and similar fungi, for which PAC2-RRPE is the preferred motif order that outperforms RRPE-PAC2 across different module attributes. These patterns are consistent both within and between fungal groups despite the fact that PAC2 is very similar to PAC and that motif order is the only thing that is variable for the intra-group comparison.

It seems that the importance of motif order extends beyond the quality of a module. The functional analyses for *S. cerevisiae, N. crassa* and *F. graminearum* suggest that for a given pair of RBA motifs, their specific order corresponds to the enrichment in a set of distinct functional terms different from the opposite module order. The two sets of annotations can be generally classified as ‘RNA-level’ and ‘protein-level’.

The presence of RBA modules can be used to search for other genes in the genome that might be involved in RBA biological process. We identified several genes in *S. cerevisiae* whose putative role in RBA is based on the presence of co-occurring RRPE and PAC motifs as well as functional annotation information and microarray expression data.

## Supporting Information

Table S1 **Best matches to RRPE and/or PAC motifs across fungal species as predicted by SCOPE.**
(XLS)

Table S2 **Module statistics for all RBA modules found in all fungal species in the study.**
(XLS)

Table S3 **Significant functional terms enriched in gene sets with two different orientations of RBA module in** ***Neurospora crassa*** **and** ***Fusarium graminearum*** **.**
(XLS)

Table S4 **Detailed functional annotation information for** ***S. cerevisiae*** **genes putatively involved in RBA based on the presence of RBA modules.**
(XLS)

## References

[1] Ashburner M, Ball CA, Blake JA, Botstein D, Butler H, et al (2000) Gene ontology: tool for the unification of biology. The Gene Ontology Consortium. Nat Genet 25: 25–29. 10802651 10.1038/75556 PMC3037419

[2] Jorgensen P, Nishikawa JL, Breitkreutz BJ, Tyers M (2002) Systematic identification of pathways that couple cell growth and division in yeast. Science 297: 395–400. 12089449 10.1126/science.1070850

[3] Cipollina C, van den Brink J, Daran-Lapujade P, Pronk JT, Vai M, et al (2008) Revisiting the role of yeast Sfp1 in ribosome biogenesis and cell size control: a chemostat study. Microbiology 154: 337–346. 18174152 10.1099/mic.0.2007/011767-0

[4] Tavazoie S, Hughes JD, Campbell MJ, Cho RJ, Church GM (1999) Systematic determination of genetic network architecture. Nat Genet 22: 281–285. 10391217 10.1038/10343

[5] Hughes J, Estep P, Tavazoie S, Church G (2000) Computational identification of cis-regulatory elements associated with groups of functionally related genes in Saccharomyces cerevisiae. J Mol Biol 296: 1205–1214. 10698627 10.1006/jmbi.2000.3519

[6] Dequard-Chablat M, Riva M, Carles C, Sentenac A (1991) RPC19, the gene for a subunit common to yeast RNA polymerases A (I) and C (III). J Biol Chem 266: 15300–15307. 1869554

[7] Liko D, Slattery MG, Heideman W (2007) Stb3 binds to ribosomal RNA processing element motifs that control transcriptional responses to growth in Saccharomyces cerevisiae. J Biol Chem 282: 26623–26628. 17616518 10.1074/jbc.M704762200

[8] Freckleton G, Lippman SI, Broach JR, Tavazoie S (2009) Microarray profiling of phage-display selections for rapid mapping of transcription factor-DNA interactions. PLoS Genet 5: e1000449. 19360118 10.1371/journal.pgen.1000449 PMC2659770

[9] Lippman SI, Broach JR (2009) Protein kinase A and TORC1 activate genes for ribosomal biogenesis by inactivating repressors encoded by Dot6 and its homolog Tod6. Proc Natl Acad Sci U S A 106: 19928–19933. 19901341 10.1073/pnas.0907027106 PMC2775034

[10] Zhu C, Byers KJ, McCord RP, Shi Z, Berger MF, et al (2009) High-resolution DNA-binding specificity analysis of yeast transcription factors. Genome Res 19: 556–566. 19158363 10.1101/gr.090233.108 PMC2665775

[11] Pilpel Y, Sudarsanam P, Church GM (2001) Identifying regulatory networks by combinatorial analysis of promoter elements. Nat Genet 29: 153–159. 11547334 10.1038/ng724

[12] Wade C, Shea KA, Jensen RV, McAlear MA (2001) EBP2 is a member of the yeast RRB regulon, a transcriptionally coregulated set of genes that are required for ribosome and rRNA biosynthesis. Mol Cell Biol 21: 8638–8650. 11713296 10.1128/MCB.21.24.8638-8650.2001 PMC100024

[13] Sudarsanam P, Pilpel Y, Church GM (2002) Genome-wide co-occurrence of promoter elements reveals a cis-regulatory cassette of rRNA transcription motifs in Saccharomyces cerevisiae. Genome Res 12: 1723–1731. 12421759 10.1101/gr.301202 PMC187556

[14] Beer MA, Tavazoie S (2004) Predicting gene expression from sequence. Cell 117: 185–198. 15084257 10.1016/s0092-8674(04)00304-6

[15] Wade CH, Umbarger MA, McAlear MA (2006) The budding yeast rRNA and ribosome biosynthesis (RRB) regulon contains over 200 genes. Yeast 23: 293–306. 16544271 10.1002/yea.1353

[16] Nguyen DH, D’Haeseleer P (2006) Deciphering principles of transcription regulation in eukaryotic genomes. Mol Syst Biol 2: 2006 0012. 10.1038/msb4100054 PMC1681486 16738557

[17] Tanay A, Regev A, Shamir R (2005) Conservation and evolvability in regulatory networks: the evolution of ribosomal regulation in yeast. Proc Natl Acad Sci U S A 102: 7203–7208. 15883364 10.1073/pnas.0502521102 PMC1091753

[18] Lavoie H, Hogues H, Whiteway M (2009) Rearrangements of the transcriptional regulatory networks of metabolic pathways in fungi. Curr Opin Microbiol 12: 655–663. 19875326 10.1016/j.mib.2009.09.015 PMC3838361

[19] Brown SJ, Cole MD, Erives AJ (2008) Evolution of the holozoan ribosome biogenesis regulon. BMC Genomics 9: 442. 18816399 10.1186/1471-2164-9-442 PMC2570694

[20] Chakravarty A, Carlson JM, Khetani RS, Gross RH (2007) A novel ensemble learning method for de novo computational identification of DNA binding sites. BMC Bioinformatics 8: 249. 17626633 10.1186/1471-2105-8-249 PMC1950314

[21] Cherry JM, Adler C, Ball C, Chervitz SA, Dwight SS, et al (1998) SGD: Saccharomyces Genome Database. Nucleic Acids Res 26: 73–79. 9399804 10.1093/nar/26.1.73 PMC147204

[22] Fitzpatrick DA, Logue ME, Stajich JE, Butler G (2006) A fungal phylogeny based on 42 complete genomes derived from supertree and combined gene analysis. BMC Evol Biol 6: 99. 17121679 10.1186/1471-2148-6-99 PMC1679813

[23] Huerta-Cepas J, Bueno A, Dopazo J, Gabaldon T (2008) PhylomeDB: a database for genome-wide collections of gene phylogenies. Nucleic Acids Res 36: D491–496. 17962297 10.1093/nar/gkm899 PMC2238872

[24] van Helden J (2003) Regulatory sequence analysis tools. Nucleic Acids Res 31: 3593–3596. 12824373 10.1093/nar/gkg567 PMC168973

[25] Mahony S, Benos PV (2007) STAMP: a web tool for exploring DNA-binding motif similarities. Nucleic Acids Res 35: W253–258. 17478497 10.1093/nar/gkm272 PMC1933206

[26] Ruepp A, Zollner A, Maier D, Albermann K, Hani J, et al (2004) The FunCat, a functional annotation scheme for systematic classification of proteins from whole genomes. Nucleic Acids Res 32: 5539–5545. 15486203 10.1093/nar/gkh894 PMC524302

[27] Hibbs MA, Hess DC, Myers CL, Huttenhower C, Li K, et al (2007) Exploring the functional landscape of gene expression: directed search of large microarray compendia. Bioinformatics 23: 2692–2699. 17724061 10.1093/bioinformatics/btm403

